# The cochlear apex demystified: Implications from synchrotron radiation phase‐contrast imaging and microscopy for cochlear implantation

**DOI:** 10.1111/joa.70001

**Published:** 2025-06-13

**Authors:** Hao Li, Rudolf Glueckert, Anneliese Schrott‐Fischer, Karin Staxäng, Hanif M. Ladak, Helge Rask‐Andersen, Sumit Agrawal

**Affiliations:** ^1^ Department of Surgical Sciences, Otorhinolaryngology and Head and Neck Surgery Uppsala University Uppsala Sweden; ^2^ Inner Ear Laboratory, Department of Otorhinolaryngology Medical University Innsbruck Innsbruck Austria; ^3^ The Rudbeck TEM Laboratory, BioVis Platform Uppsala University Uppsala Sweden; ^4^ Department of Otolaryngology—Head and Neck Surgery Western University London Ontario Canada; ^5^ Department of Medical Biophysics Western University London Ontario Canada; ^6^ Department of Electrical and Computer Engineering Western University London Ontario Canada

**Keywords:** 3D rendering, cochlea, cochlear implant, human, synchrotron‐PCI

## Abstract

Due to the complex organization of the human cochlear apex, further analysis of the tonotopic relationship between the organ of Corti (OC) and spiral ganglion (SG) is required in relation to cochlear implantation. In this study, the human SG nerve fiber organization and ultrastructure were assessed using semi‐thin light microscopy sectioning and three‐dimensional (3D) synchrotron radiation phase‐contrast imaging (SR‐PCI). A fresh human temporal bone underwent high‐resolution SR‐PCI with a dual‐detector system. Orthogonal sectioning, cropping, and tissue segmentation were used to create high‐resolution 3D reconstructions. Peripheral dendrites were traced from the basilar membrane to the SG, and a tonotopic map was constructed using Greenwood's function. Results were compared and validated against novel high‐resolution microscopy data of a sectioned human cochlea. Only the basal and initial middle turn of the cochlea displayed a well‐defined Rosenthal's canal (RC), and after 450 degrees, this converged into a central modiolar space. The OC and SG tonotopic maps remained closely aligned for angular depths up to approximately 650 degrees, after which the SG frequencies became significantly more spatially compact relative to the OC. In the central modiolus, the apical 1.37 mm of the SG contained over four octaves of tonotopic representation. In comparison, the compressed apical SG represented 9.6 mm of the OC (28% of the overall length) over the same tonotopic range. These results were validated with microscopy, which revealed that this apical SG contained around 8000 neurons and represented 960 inner hair cells along the OC. This is the first study to present the detailed cellular organization and 3D tonotopic arrangement of the human SG within the central modiolus. For low frequency stimulation, rate‐based coding may be required to augment tonotopic mapping in the compressed SG regions. In addition, the OC tonotopic map has significantly less compression and could potentially be targeted directly for place‐based coding.

## Graphical abstract

This study is the first to present the detailed cellular organization and three‐dimensional (3D) tonotopic arrangement of the human organ of Corti, spiral ganglion, and central modiolus using synchrotron radiation phase‐contrast imaging (SR‐PCI) with matched histological cross‐sections. The results, including dimensions of octave bands and rotational angles, are relevant for optimizing electrical stimulation of the human cochlear nerve in cochlear implantation.

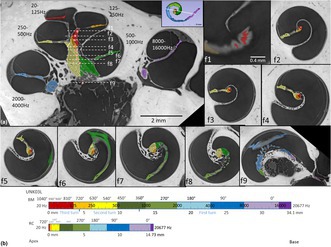

## 1 INTRODUCTION

Patients with profound sensorineural hearing loss (SNHL) can be treated with a cochlear implant (CI) that electrically excites a population of neurons within the cochlea. The mechanisms that underlie how and where these neurons are stimulated are still poorly understood. In order to provide pitch perception to recipients, CI electrode arrays must be tonotopically programmed with a frequency‐to‐place map, whereby high frequency information is presented in the basal portion of the cochlea and low frequency information is presented in the apical portion of the cochlea. CIs have been traditionally programmed using a generalized frequency‐to‐place map, where frequency information is distributed to electrode contacts using a default map irrespective of patients' anatomy or postoperative implant location within cochleae. This generalized frequency‐to‐place mapping strategy can result in frequency‐to‐place mismatch, where sound information is presented to the wrong tonotopic frequency location. In addition, the anatomy of the cochlear apex is complex, and the tonotopic mapping of the spiral ganglion (SG) in this area is poorly understood (Deman et al., 2009; Gani et al., 2007; Landsberger et al., 2014).

Recently, there has been a growing body of research on individualized CI frequency‐to‐place mapping. Individualized mapping approaches use information on patients' anatomy and post‐operative electrode contact locations to develop a patient‐specific frequency‐to‐place map. Such maps have been developed to model tonotopic distributions at the level of the organ of Corti (OC) as well as the more central neuron cell bodies at the spiral ganglion (SG). Tonotopically accurate low‐frequency stimulation may be essential for improved CI performance in speech and music. Frequency‐place matching is also important to develop stimulation strategies using temporal coding for the low‐frequency region with electrodes placed in the second cochlear turn (Dhanasingh & Hochmair, 2021; Hochmair et al., 2015; Landsberger et al., 2016). It may also be constructive to target preserved apical neurons in CI stimulation since neural degeneration is often more severe in the basal cochlea in hearing‐impaired individuals, with a diminished number of peripheral processes, and often associated with altered evoked compound action potential (ECAP)‐amplitude and thresholds levels (Felder et al., 1997; Felder & Schrott‐Fischer, 1995; Felix et al., 1992; Linthicum & Fayad, 2009; Nadol, 1997; Nadol et al., 1989). Studies on the tonotopic relationship of the OC and SG in the cochlear apex are, however, limited, particularly in three‐dimensional (3D) data. Accurate 3D cochlear imaging datasets are required to accurately analyze OC and SG tonotopy and inform frequency‐place mapping.

Several imaging methods have been used to model the human inner ear (Counter et al., 2003; Elfarnawany et al., 2017; Lareida et al., 2009; Poznyakovskiy et al., 2008); however, few studies have focused on the cellular organization of the human spiral ganglion, particularly the apical mid‐modiolar region. Furthermore, most studies are based on experimental animal data or human tissue processed after death with incomplete structural preservation and limited optical resolution in microscopic investigation.

We recently analyzed the cytoarchitecture of the human cochlear partition at different frequency locations using high‐resolution microscopy in a uniquely preserved human cochlea (Giese et al., 2024). Plastic‐embedded sections were analyzed using light and transmission electron microscopy. Frequency locations were estimated using analogous synchrotron radiation phase‐contrast imaging (SR‐PCI) data of a fixed, unstained adult human temporal bone. The goal of the present study was to use an equivalent analysis of the modiolus and auditory nerve in a temporal bone specimen to investigate the human auditory nerve, including its fiber organization and tonotopy distribution in the central modiolus and meatal aperture, and to relate these insights to CI stimulation. A 3D tonotopic map was created with frequency positions of the OC and SG through dendrite tracing. Frequencies were assessed by nonlinear least squares fitting of the Greenwood function (Greenwood, 1961). Correlations between high‐resolution light and electron microscopy and synchrotron imaging were used to assess the 3D arrangement of the helically shaped spiral ganglion, central axons, and peripheral dendrites for the first time.

## 2 MATERIALS AND METHODS

### 2.1 SR‐PCI sample preparation, imaging, and analysis

The SR‐PCI technique used in the present investigation has been previously described (Elfarnawany et al., 2017). The sample was scanned using the Bio‐Medical Imaging and Therapy (BMIT) 05ID‐2 beamline at the Canadian Light Source, Inc. in Saskatoon, Saskatchewan, Canada. SR‐PCI was used to obtain a 3D CT scan and reconstruction of the specimen with an isotropic voxel spacing of 9 μm. No staining, sectioning, chemical dehydration, or decalcification was performed on the specimen.

For 3D assessment of the cochlear partition, peripheral axons (dendrites), and SG, structures were segmented (i.e., delineated and color labelled) manually in each SR‐PCI CT slice using the open‐source medical imaging software 3D Slicer (www.slicer.org, version 5.0.3). Approximately 1400 SR‐PCI CT slices were utilized to segment the BM, peripheral axons (dendrites), and SG. The process of segmenting these structures in SR‐PCI data has been previously described (Li et al., 2021). For qualitative validation of the anatomical observations made in the SR‐PCI data, archival mid‐modiolar histologic microscopy images prepared for light and electron microscopy including immunohistochemistry were used (Liu et al., 2021; Pamulova et al., 2006; Tylstedt et al., 1997).

The BM was manually traced in 3D in the SR‐PCI CT scan to obtain anatomically accurate landmarks from the basal‐most point of the BM in the cochlear hook region to the apical termination of the BM in the helicotrema. Landmarks were placed along the estimated center path of the OC in the BM segmentation. The complete BM length and proportional BM length associated with each landmark were calculated. Greenwood's function, which relates proportional distances along the BM to tonotopic frequencies, was used to estimate the OC tonotopic frequency at each manually placed landmark (Greenwood, 1961). Peripheral dendrites were traced from the BM to the RC at each BM tonotopic region in 3D to obtain SG tonotopic frequency landmarks. Individually tracing peripheral dendrites allowed for precise tonotopic representation of the SG, particularly in the regions where peripheral axons (dendrites) follow nonradial trajectories from the BM to RC. Due to the spatial resolution of SR‐PCI, cellular details within the SG were not discernible; however, the paths of the dendrites tracing from the BM to the bony conduit of the RC were visible.

In order to analyze the relationship between angular length along the cochlea and OC and SG tonotopic frequency, the angular depth of each OC and SG frequency coordinate was obtained. A reformatted SR‐PCI slice was generated through the mid‐modiolar axis of the cochlea. The reformatted mid‐modiolar image slice was rotated about the mid‐modiolar axis through the length of the cochlea. With 0 degrees defined as the center of the round window, the angular depth of each previously determined OC and SG tonotopic coordinate was measured.

For the OC and SG, the tonotopic frequencies were plotted relative to the linear length (measured in millimeters) and angular length (measured in degrees) of the structures. In addition to the relationships between length and tonotopic frequency, a secondary analysis was performed to estimate the rate of change of tonotopic frequency throughout the length of the OC and SG relative to both linear and angular length. To relate the tonotopic rate of change to relative, perceptual changes, the logarithm of the rate of change was calculated to convert the frequency changes to measures of octaves.

### 2.2 Histological sample preparation and imaging

Light microscopy (LM) and transmission electron microscopy (TEM) data were obtained from one human cochlea earlier described by Giese et al. (2024). It was removed from a patient with normal hearing during trans‐cochlear surgery due to petroclival meningioma. The surgery was performed as a two‐stage operation with facial nerve rerouting postero‐inferiorly and total petrosectomy followed by complete tumor removal. The operation time was approximately 15–20 h, performed by oto‐ and neurosurgeons. After facial nerve rerouting, the cochlea was dissected out (adding an additional 10 min to the total surgery time) and placed immediately in phosphate buffered 2% glutaraldehyde in 0.05 M sodium phosphate and 13.3% fluorocarbon to elevate oxygenation. It was decalcified in 0.1 M phosphate‐buffered Na‐EDTA (sodium‐ethyldiaminetetraaceticacid) for 6 weeks followed by postfixation in 1% OsO4 in phosphate buffer. The cochlea was dehydrated in graded ethanol then placed in propylene oxide and embedded in epoxy resin. The cochlea was divided into two halves at the apical rim of the round window and across the modiolus, and a complete mid‐modiolar section was made for LM and TEM. Serial semi‐thin sections (1 micron thick) were stained with toluidine blue for LM to conceptualize the principal organization of the cellular morphology of the central modiolus. Sections were photographed in an Olympic CX23 binocular microscope. A complete series of semi‐thin sections could not be retrieved. The level of sectioning through the cochlea was assessed by morphological characteristics of the OC followed by thin sectioning of areas of interest. These sections were stained in lead citrate and uranyl acetate and examined at 80 kV with a Tecnai™ G2 Spirit TEM (Thermo Fisher/FEI Company, Eindhoven, NL). Images were captured with an ORIUS™ SC200 CCD camera (Gatan Inc., Pleasanton, CA, USA) using Gatan Digital Micrograph software. Some images of the SG were published in an earlier investigation (Liu & Rask‐Andersen, 2022).

The topographic cell anatomy and distribution of central and peripheral axons in the central modiolus were assessed. Thin sections were made at strategic levels and contrasted for TEM, as earlier described (Giese et al., 2024).

### 2.3 Comparison of histologic sample and SR‐PCI sample

Construction of analogous SR‐PCI and histological sections made it possible to compare the anatomical details, with the goal of understanding neurite and ganglion cell organization in the central modiolus. SR‐PCI did not allow for thorough differentiation between unstained SG soft tissue and discrete ganglion cells. Comparisons made in histological sections revealed that nerve fascicles ran in a network of bony septa, and these could then be resolved in SR‐PCI. The ganglion cell mass was thereby well distinguished from nerve fascicles and reconstructed in SR‐PCI in the central modiolus.

## 3 RESULTS

### 3.1 Image analysis and tonotopic mapping

**FIGURE 1:**
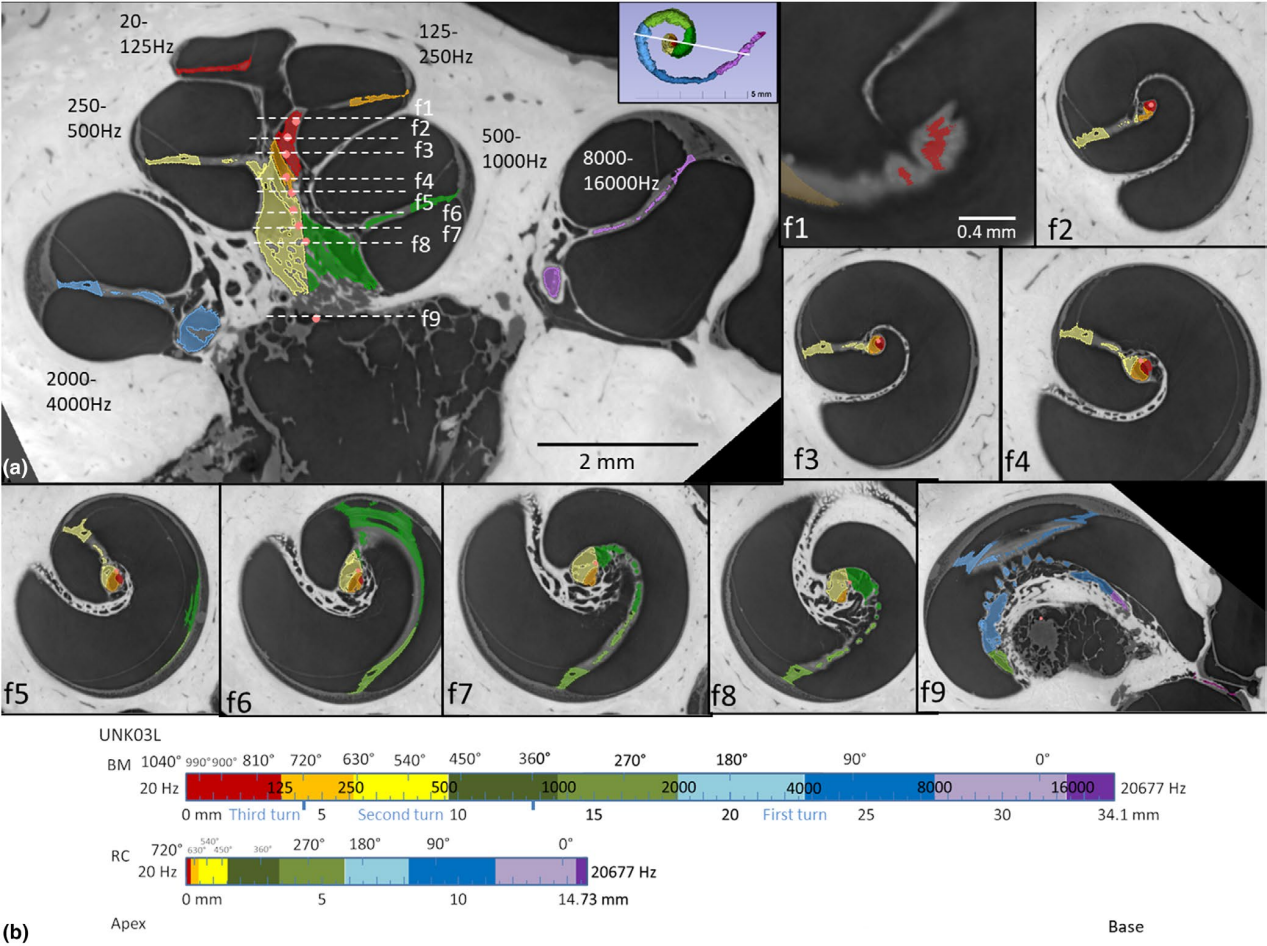
(a) SR‐PCI, mid‐modiolar section with segmentation of the organ of Corti, spiral lamina, and nerve elements in specimen UNK03L. Serial axial cross‐sections of the cochlea show the modiolus at different levels using the “jump slicer” technique (f1–f9). Matching microscopic cross‐sections suggest apical ganglion cell bodies reach levels f2 and f3. (b) Dimensions of octave bands and rotational angles for tonotopic frequencies of the basilar membrane (BM) and spiral ganglion (SG) are shown. Angular rotations are calculated from the mid‐point of the round window.

**FIGURE 2:**
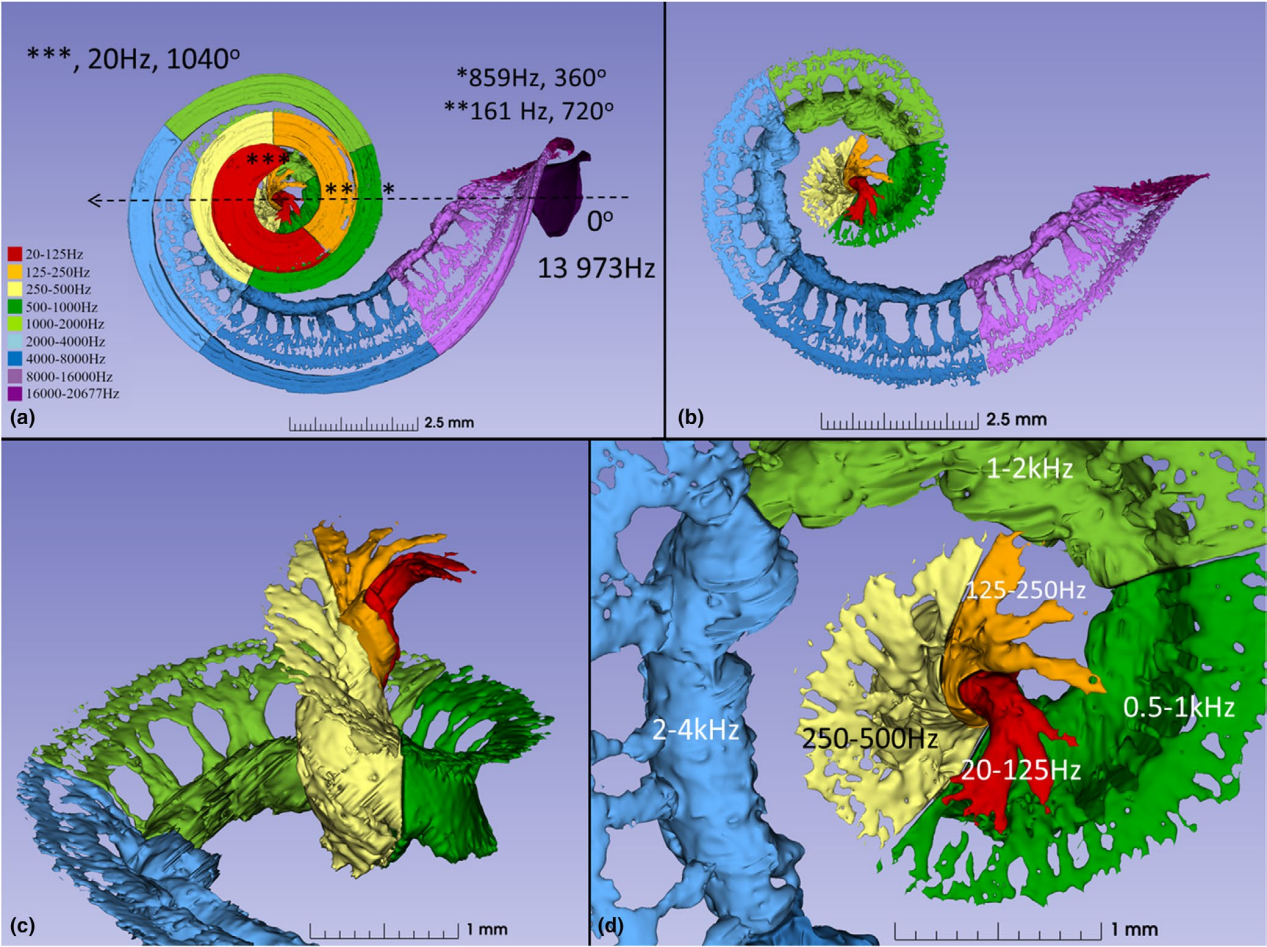
(a, b) SR‐PCI, segmentation and 3D rendering of the human cochlea (left ear). The basilar membrane/limbus and dendrites were traced to Rosenthal's canal and frequency coordinates were created using the Greenwood function. The diameter A extends from the mid‐portion of the round window (0°, 13,973 Hz) to the mid‐modiolar axis. Turn one ended at 859 Hz, turn two ended at 161 Hz, and turn three ended at 20 Hz. (c, d) Basally, dendrites extend radially within columns to reach Rosenthal's canal. At approximately 650 degrees (230 Hz), the apical dendrites start to rotate clockwise.

**FIGURE 3:**
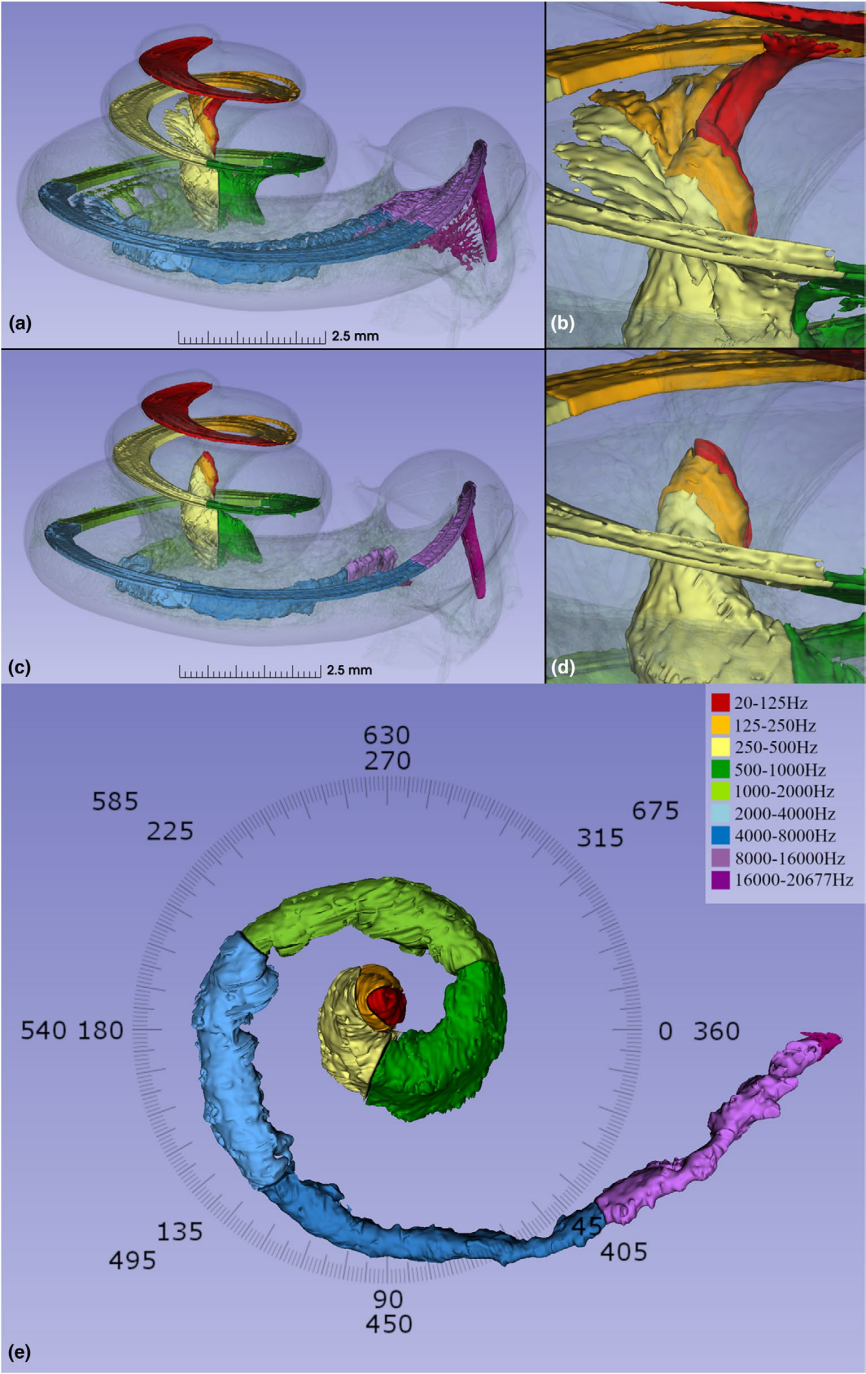
SR‐PCI, segmentation, and 3D rendering of the specimen with (a, b) and without (c, d) dendrites. (e) Spiral ganglion channel consists of an outer part named Rosenthal's canal that reaches approximately one and a quarter turns (450°, ~500 Hz) and a central more twisted portion (here named the central modiolus) reaching two turns (720°).

**FIGURE 4:**
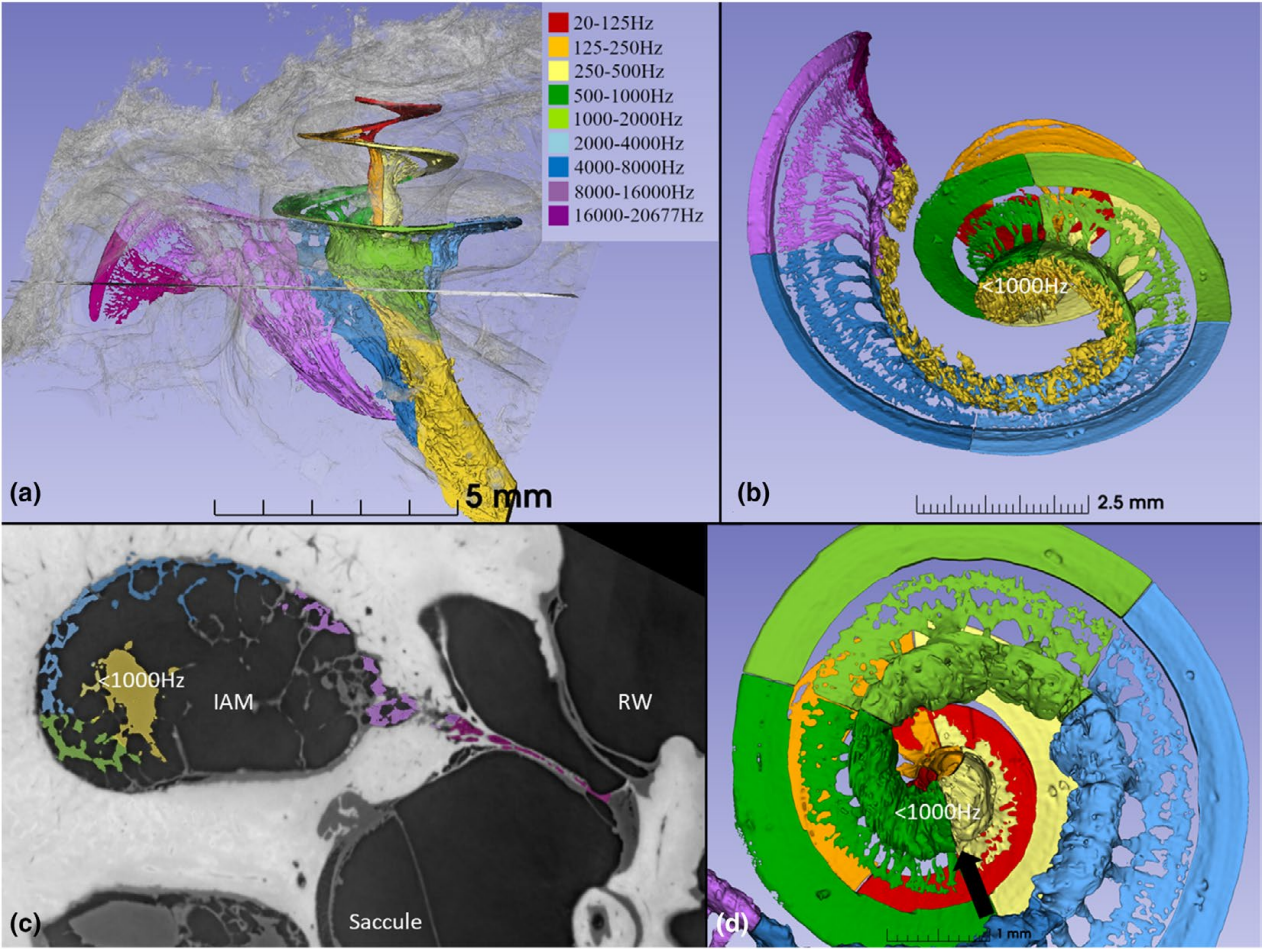
(a) 3D rendering of the left human cochlea and acoustic nerve (superior view, section line at meatus is shown in c). (b) Meatal view displays the comma‐shaped acoustic nerve (yellow), consisting of a central trunk and a “tail” of nerve fascicles entering through several bony foramina. (c) Cross‐sectioned cochlear nerve at the internal acoustic meatus (IAM) (section level shown in a). Tonotopic distribution of various nerve fascicles are seen with the central trunk (yellow) containing fibers tuned below 1000 Hz. (d) Meatal view with removed central axons shows the boundary between Rosenthal's canal and central modiolus at 450 degrees (arrow). RW, round window.

SR‐PCI CT scan slices, segmentations, and respective 3D visualizations of the frequency‐separated BM, peripheral dendrites, and RC are illustrated in a left ear in Figures 1, 2, 3, 4. Serial axial cross‐sections of the cochlea showed the modiolus at different levels. The cochlea had a total BM length of 34.10 mm: 21.38 mm in the first turn, 8.37 mm in the second turn, and 4.35 mm in the apical turn (Figure 1). Matching microscopic cross‐sections suggested that apical ganglion cell bodies reached section levels f2 and f3, as depicted in Figure 1. A well‐defined RC was observed only in the basal and first part of the second turn of the cochlea. The SG had a total curvilinear length of 14.73 mm: 11.95 mm in the first turn and 2.78 mm in the second turn. The RC length was 13.36 mm, extending beyond an angular depth of 450 degrees, reaching up to 720 degrees. The SG extended approximately 1.37 mm into a central modiolar space representing tonotopic regions below 500 Hz. This represented 9% of the total SG length.

The BM/limbus and dendrites were traced to RC and frequency coordinates created using Greenwood's formula. Turn one ended at 859 Hz, turn two ended at 161 Hz, and turn three ended at 20 Hz (Figure 2). The studied cochlea had a BM angular length of 1040 degrees, and a SG angular length of 720 degrees. At 650 degrees, the apical dendrites started to rotate clockwise. SR‐PCI, segmentation, and 3D rendering with and without dendrites are shown within the cochlear shell in Figure 3, where the central modiolus and SG reached two turns.

3D rendering of the intra‐meatal acoustic nerve showed it was shaped as a “comma‐sign,” with a central trunk containing axonal fibers tuned below 1000 Hz (Figure 4a,b). The central trunk and the “tail” of nerve fascicles entered several bony foramina. The cross‐sectioned internal acoustic meatus (IAM) showed the tonotopic distribution of nerve fascicles and central nerve trunk containing fibers tuned below 1000 Hz (Figure 4c). The central trunk met Rosenthal's canal at the central fundus, as shown in Figure 4d.

**FIGURE 5:**
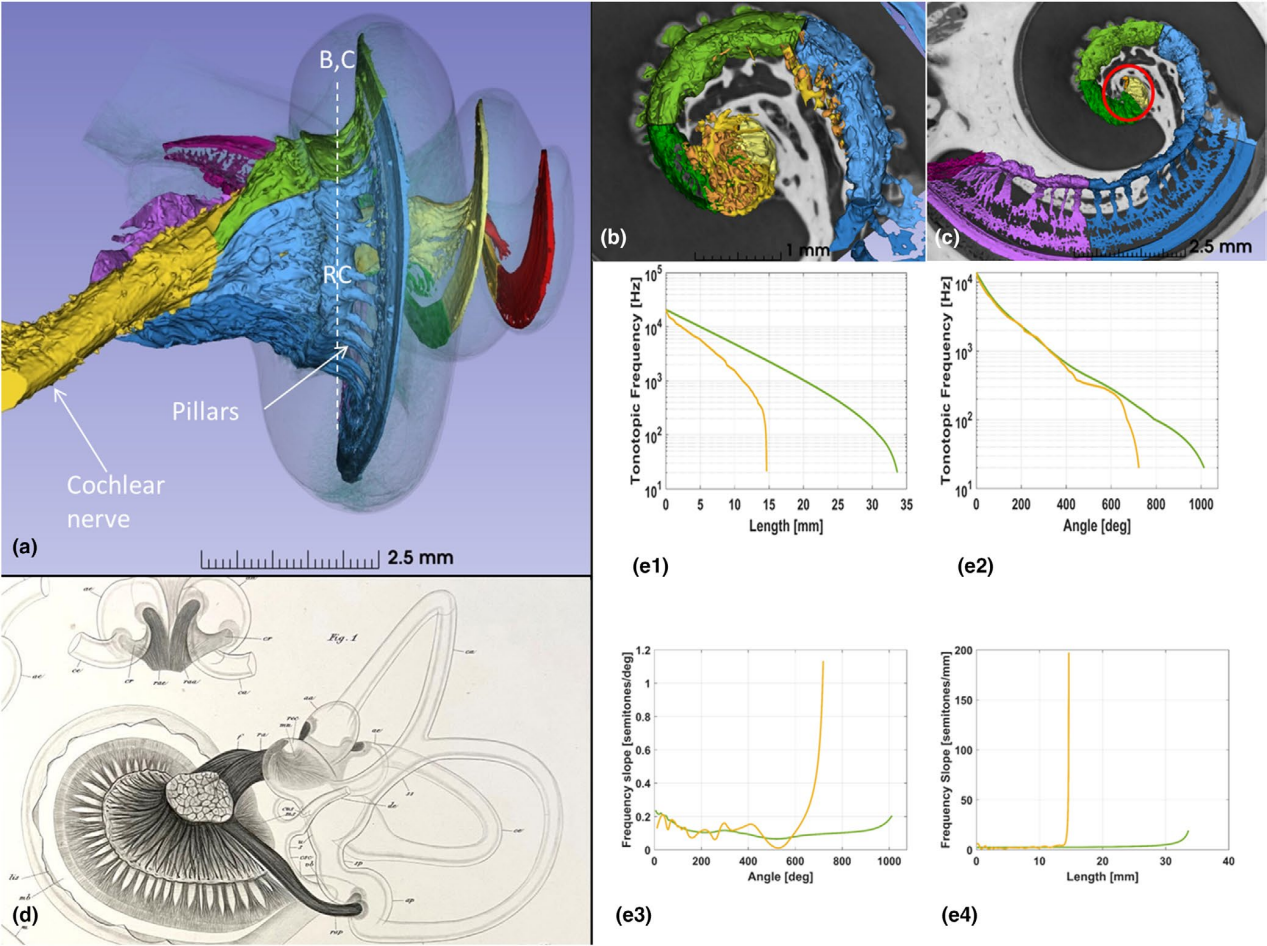
(a) Segmented left cochlea with multi‐colored frequency‐based delineation of the nerve components and basilar membrane (medial view). Axons separate from the main trunk in the lateral internal acoustic meatus forming a rotated “drape” before reaching the Rosenthal's canal (RC). Interrupted line (b, c) shows the level of sections demonstrated in figures (b) and (c). (b) Axial 3D rendering of the central nerve truck (yellow) reaching the fundus of the internal acoustic meatus and Rosenthal's canal. (c) (Red circle). Same section level as in b demonstrating the union of the Rosenthal's canal (500–1000 Hz) and central modiolus (250–500 Hz). (d) Gustaf Retzius drawing (1884) of brush‐like arrangement of meatal cochlear nerve fibers in a 5‐month‐old human embryo. (e1–e2) Tonotopic relationships of the OC and SG. Tonotopic frequencies are plotted relative to the curvilinear length of the respective structure (e1) and the angular length of the respective structure (e2). (e3–e4) Tonotopic rates of change of the OC and SG. The OC (green) and SG (yellow) tonotopic frequency slopes are plotted relative to the angular length of the respective structure (e3) and the curvilinear length of the respective structure (e4), Table 1.

**FIGURE 6:**
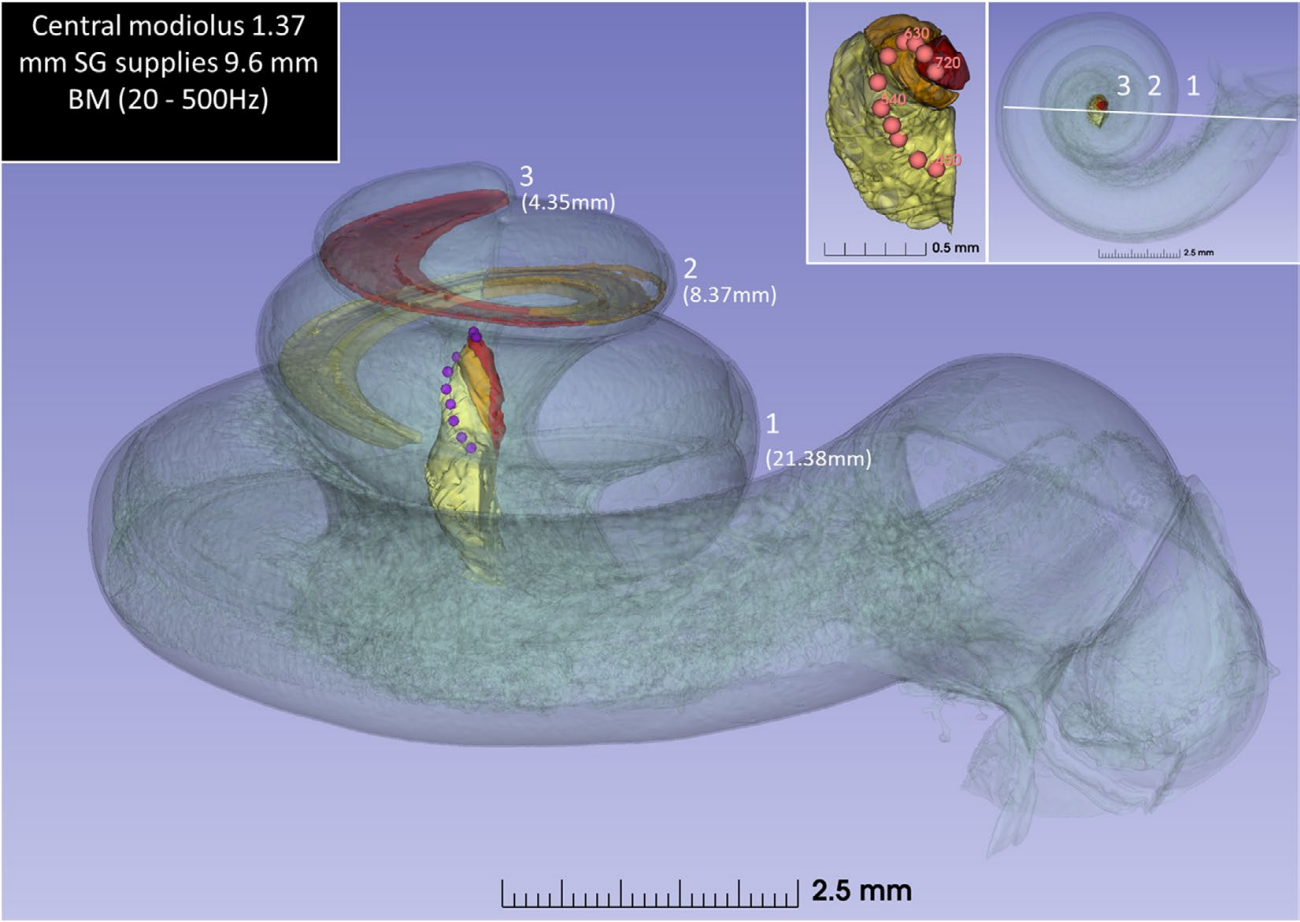
3D rendering of the human central modiolus forming a tight spiral (three quarters of a turn, 450°–720°) and containing neurons innervating hair cells in the apical 9.6 mm (28% of the total BM curvilinear length, frequencies 20–500 Hz, angular rotation 450°–1040°). The curvilinear lengths of the three cochlear turns are shown in millimeters. Our scanning electron microscopy data and earlier analyses show that this region contains around 100 IHCs per mm; therefore, these neurons may supply approximately 960 inner hair cells. BM, basilar membrane; SG, spiral ganglion.

A medial view of the left acoustic nerve is shown in Figure 5a. Basally, dendrites extended radially within pillars or columns to reach the spiral lamina. Separated axons in the IAM formed a rotated “drape” reaching the circular Rosenthal's canal. The cross‐sectioned IAM is shown with and without central axons in Figure 6b,c. Central modiolar axons were seen to reach both the 250–500 Hz (yellow) and the 500–1000 Hz (green) SG regions. A drawing of a right human acoustic nerve produced by the Swedish anatomist Gustaf Retzius is shown in Figure 5d (Retzius, 1884).

**TABLE 1:** Summary of organ of Corti (OC) and spiral ganglion (SG) frequencies, and the respective absolute semitone difference between the structures at 90‐degree increments.

	0	90	180	270	360	450	540	630	720	810	900	990
OC frequency (Hz)	13,876	4951	2590	1493	832	519	363	245	152	91	52	26
SG frequency (Hz)	13,996	5119	2645	1467	834	463	336	202	20	N/A	N/A	N/A
Absolute semitone difference	0.1	0.6	0.4	0.3	0.0	2.0	1.3	3.3	35.1	N/A	N/A	N/A

The relationship between curvilinear length and angles and tonotopic frequencies for the BM and SG is presented in Figure 5e1,e2. The OC and SG frequency maps remained closely aligned for angular depths up to approximately 650 degrees, at which point the most notable divergence occurred and the SG frequencies became more spatially compact relative to the OC. To assess the relative spatial resolution of frequency, the logarithm of the rate of change of tonotopic frequencies was calculated. This provided an estimate of the number of semitones per millimeter and degree along the OC and SG (Figure 5e3,e4). The compression of tonotopic frequencies in the apical SG can be observed by both the angle–frequency relationship and the rate of change of frequency.

The length of the BM supplied by neurons in the central modiolus corresponded to 9.6 mm (Figure 6). It represented 28% of the total basilar membrane curvilinear length (450 degrees–1040 degrees), constituting frequencies 20–500 Hz. In another human specimen with normal hearing, this part of the cochlea contained approximately 960 IHCs (100 IHCs/mm), as shown by SEM (Rask‐Andersen et al., 2017).

### 3.2 Cell organization and anatomy of the central modiolus

**FIGURE 7:**
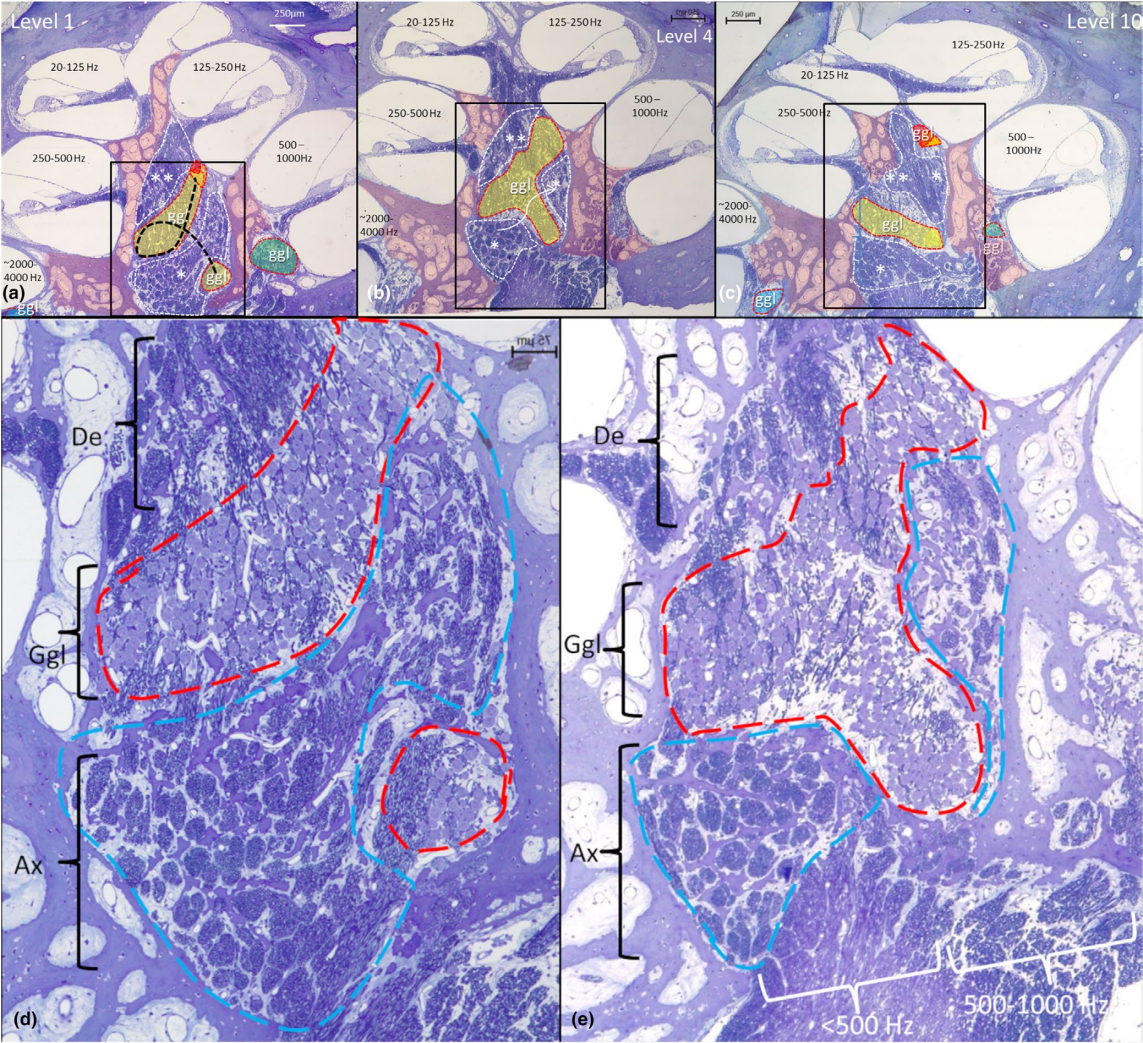
(a–c) Light microscopy of high‐resolution semi‐thin sections (<1 μm) of the mid‐modiolar region in a human cochlea at three different section levels (1, 4, and 10). Section level 1 corresponds to the SR‐PCI section shown in Figure 1. Labeled areas encircled by red broken lines represent populations of spiral ganglion cells (Ggl), while those bounded by white broken lines (*) represent central and peripheral axon bundles. Nerve fascicles are separated in a bony trabecular network forming a meshwork. The bony modiolar wall is shown in pink. Framed areas are magnified in d and e. (d, e) Higher magnification of mid‐modiolar regions shown in a (level 1) and b (level 4). The mid‐modiolar space is densely populated by axons (Ax), dendrites (De) and spirally arranged nerve fascicles arranged centrally of the tightly packed spiral ganglion (Ggl) cell bodies. Axon fascicles are outlined by a blue dashed line and the ganglion cells by a red dashed line. There are only small capillary vessels present among mid‐modiolus soft tissue. (e) The central nerve trunk consists of two parts innervating the 20–500 Hz and the 500–1000 Hz regions.

An earlier documented human cochlea offered unique opportunities to analyze cell organization of the human SG (Giese et al., 2024). Mid‐modiolar sections equivalent to the corresponding frequency‐separated segmentations in the SR‐PCI data (as shown in Figure 1) are illustrated in Figure 7a–e.

**FIGURE 8:**
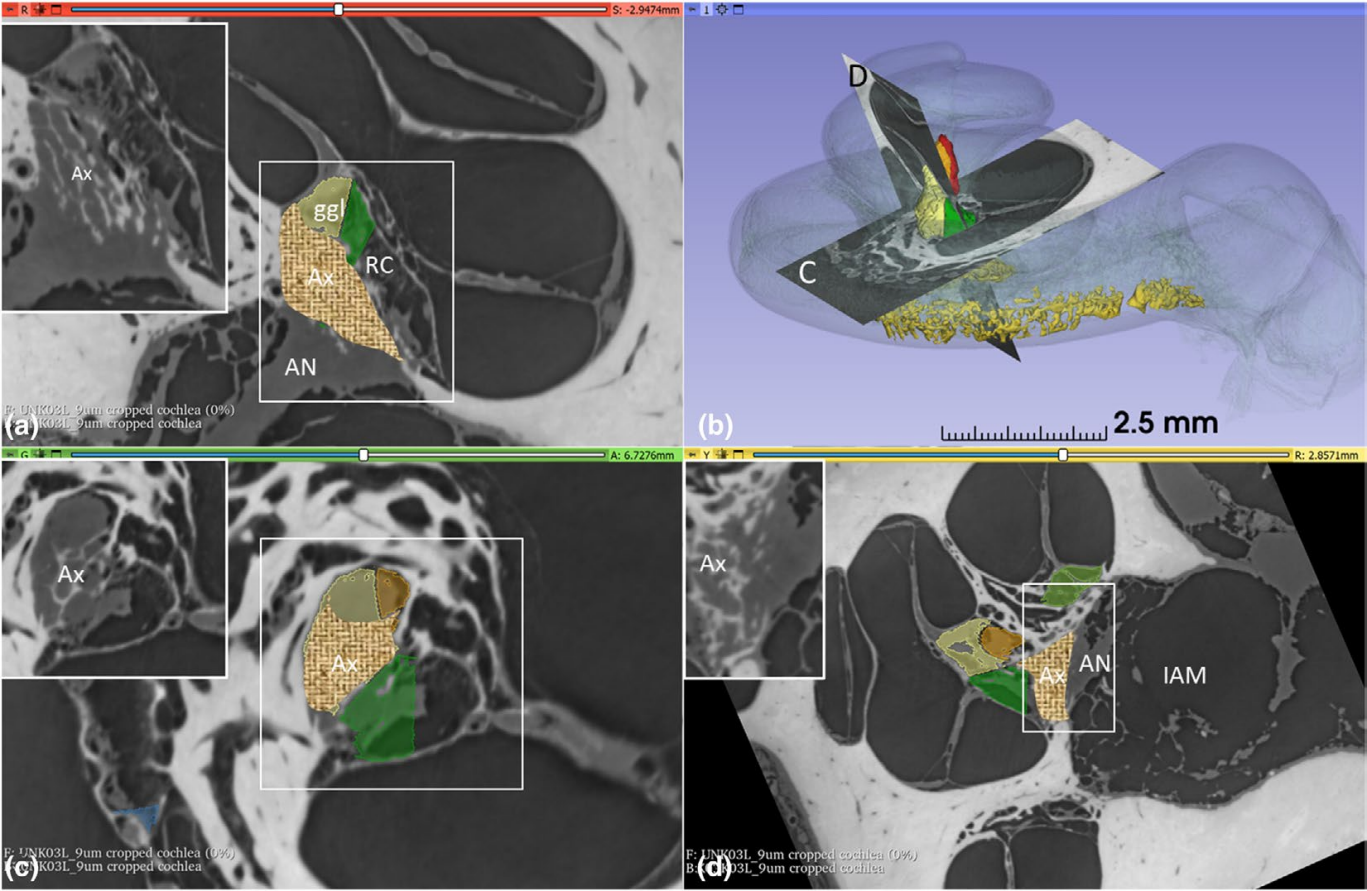
SR‐PCI and 3D rendering of the mid‐modiolar space. (a) Section shows the central nerve trunk and axons (Ax) entering a bony trabecular meshwork before reaching the coalition between Rosenthal's canal (RC) and ganglion cells (ggl) in the central modiolus. (b) demonstrates orthogonal sections in (c) and (d). AN, acoustic nerve; IAM, internal acoustic meatus.

The central modiolus consisted of a rhomboid‐pyramidal space containing a density of central axons, spiral ganglion cells, and peripheral axons. Its height was 1.95 mm, with a basal diameter of 0.96 mm and a volume of 0.47 mm^3^. It was margined by bony walls containing a plexus of mostly thin‐walled blood vessels. The ganglion cell region comprised many capillaries but no larger vessels. The SG formed a helix‐shaped cylinder with an approximate length of 1.51 mm and a diameter of approximately 200 microns. The ganglion cells were reached by central axons entering through a parallel and twisted bony trabecular meshwork, which contained nerve fascicles originating from the central nerve trunk and entering the fundus perforations. Axon fascicles reached the infero‐medial poles of the ganglion cells, while peripheral axons exited supero‐laterally. Ganglion cells faced the medial wall of the scala tympani in the upper turn (125–250 Hz region; f2 in Figure 1a). Ectopic neurons occasionally appeared in the trabecular meshwork and IAM. The axon fascicles formed a trabecular meshwork, which was then confirmed in synchrotron imaging (Figure 8). The density of cell bodies was higher in the central modiolus than in the RC.

**FIGURE 9:**
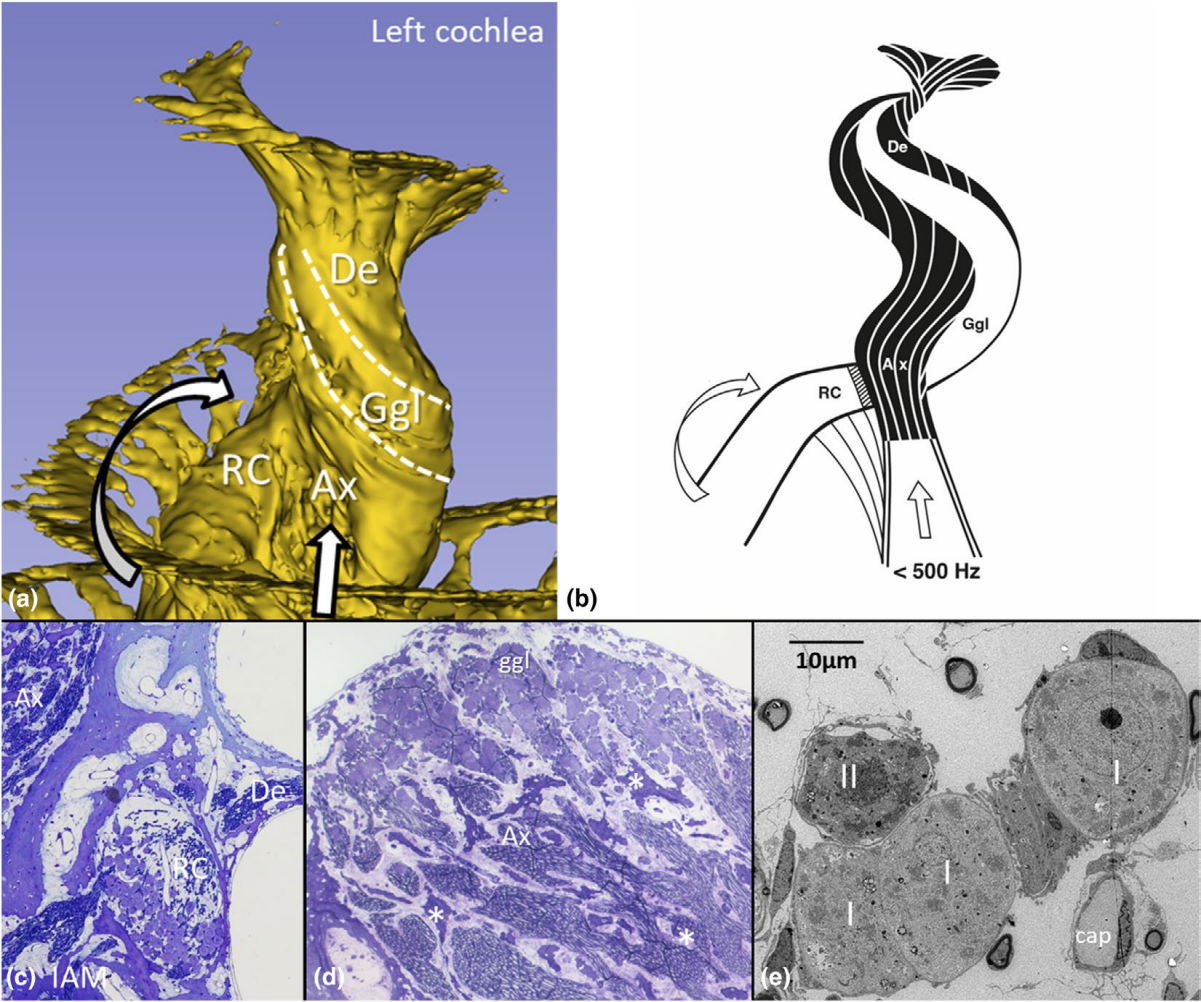
(a) SR‐PCI 3D rendering of the central mid‐modiolar region and the coalition of RC and the mid‐modiolus and central nerve trunk tuned for frequencies below 500 Hz. (b) Artist's rendition of the organization of the human central modiolus. (c) Light microscopy of the sectioned RC at the entrance into the central modiolus. (d) Light microscopy of the cross‐sectioned mid‐modiolar space with spiral ganglion cells located peripherally and axon trabecular network (*) centrally. (e) Transmission electron microscopy of type I and II ganglion cells in the central modiolus. Cap, capillary; Ax, axons; IAM, internal acoustic meatus; De, dendrites; Ggl, spiral ganglion cell bodies.

SR‐PCI 3D rendering, LM and TEM of the central mid‐modiolar region show the amalgamation of RC, mid‐modiolar space, and the central nerve trunk tuned for frequencies below 500 Hz (Figure 9).

## 4 DISCUSSION

Various imaging techniques such as high‐resolution micro‐computed tomography (μCT) have been used to create 3D reproductions and finite element models to optimize strategies for electric stimulation with CIs (Bai et al., 2019; Croner et al., 2022; Potrusil et al., 2020). These studies may be used as a basis for place‐frequency representation of CI electrode arrays to selectively stimulate the low frequency nerve fibers in the apical cochlea. However, they are hindered by the complexity of the neural pathways and microstructures housed within the human cochlea. There is a particular paucity of knowledge regarding the cell anatomy and 3D topography of the central modiolus and apical region. In this region, nerve cell bodies are packed together with central and peripheral axons, whose close relationship may hinder segregated nerve fiber activation by electric stimulation. In the present study, SR‐PCI data of an intact, unstained human cochlea allowed for visualization, measurement, and analysis of the OC and SG tonotopy, including the apical region, at a level of detail not previously achieved. Utilizing 3D SR‐PCI data, the BM and RC were segmented in each 2D slice using color labelling in anatomic regions. Additionally, the paths of peripheral axons between the OC and SG pathways were traced and segmented in order to determine the tonotopic distribution of the SG.

As observed in the SR‐PCI scan and confirmed in previous histological reports, RC is a distinct conduit for the first approximately 450 degrees of angular depth. Beyond this point, the central modiolus becomes a common area where the SG cell bodies corresponding to all angular depths beyond 450 degrees are in a shared modiolar space. Tonotopic regions originating on the BM remain spatially separated through peripheral axons into the central modiolus, and despite the common space in the apex, the SG cell bodies and nerve fibers remain tonotopically organized. This may be attributed to the bony trabecular network allowing even the most apical axonal fascicles to be tonotopically separated as they reach corresponding cell soma. There is limited bony separation between the tonotopically organized SG cell bodies, indicating that there may be additional considerations required for electrical stimulation in this region.

### 4.1 Innervation density of the apical cochlea

The total number of SG cells in the human cochlea is stated to be around 30,000–35,000 (Guild et al., 1931; Hinojosa et al., 1985). The number of cells and nerve fibers supplying different turns of the cochlea has been shown to vary, with the concentration of cells believed to be greatest in the upper basal and lower middle coil, with a decrease at either end of the cochlea (Felix et al., 1990; Guild et al., 1931; Hinojosa et al., 1985; Ishiyama et al., 2011; Miura et al., 2002; Nadol, 1988; Otte et al., 2015; Pollak et al., 1987; Rasmussen, 1940; Retzius, 1884; Spoendlin & Schrott, 1988, 1989; Wever, 1949). Large variations have also been shown in the apical 5 mm and beyond (apical 7.88 mm), with a mean number of 6234 cells reported by Hinojosa et al. (1985), and 3267 cells reported by Nadol (1988). These variances may be explained by effects of age and cochlear length; by different analytic techniques used; and by difficulties in quantifying neurons in the compressed apical region.

A literature review conducted by Dhanasingh et al. (2020) revealed that 25.8% of the total number of SG cell bodies were believed to be located in the apical segment, reaching the apical 10 mm of the cochlea (angular depth of 400 degrees to the helicotrema). According to Nadol (1988), the apical region, distal to 26 mm OC length, housed approximately 10% of the total SG cell population in normal‐hearing subjects. Otte et al. (2015) found that an apical SG length of 1.7 mm served 10 mm length of the OC (22–31 mm) and held 28% of the total SG cells with a best frequency range below 900 Hz. The authors concluded that 3000 SG cells were needed in this region to provide useful speech discrimination.

Moreover, studies have reported that IHCs are significantly less innervated in the apex compared to the lower middle turn (Guild et al., 1931; Spoendlin & Schrott, 1989). Our knowledge of the number of IHCs in the human cochlea derives from early studies by Waldeyer (1872), Krause (1876), and Retzius (1884), and was later refined by Bredberg (1968), Wright (1983), Wright et al. (1987), Úlehlová et al. (1987), and Spoendlin and Schrott (1988, 1989, 1990) using enhanced surface techniques, with reported values ranging from 2936 to 3630. According to Nadol (1983), each IHC receives approximately 6–8 different nerve terminals, and each terminal possesses a variable number of synaptic contacts in the basal (10 mm region) and middle turns (26 mm region). Spoendlin and Schrott (1989) found that IHC density increased steadily from base to apex, with around 50 cells/mm at the base and over a 100 cells/mm at the apex. The authors found 1400 lamina fibers per mm in the 0.5–0.7 region of the BM (divided into 10 segments from base to apex), resulting in 15 nerve fibers per IHC. In the apical turn, they found 400 fibers per mm, corresponding to 3–4 fibers per IHC. However, in one 49‐year‐old male with a normal audiogram and a total of 33,462 cochlear nerve fibers, reanalysis of the original counts revealed 10.9 and 6.7 lamina nerve fibers per IHC in the 0.8 and 0.9 regions, respectively, which is only slightly less than in the lower second turn (Spoendlin & Schrott, 1990, Annelies Schrott‐Fischer, personal communication). Our earlier SEM data from a normal hearing individual revealed a segmental number of approximately 110–120 IHCs per mm in the cochlea (Rask‐Andersen et al., 2017). The studied region ranged from high frequencies to around 200 Hz. This is in accordance with that described by Bredberg (1968) and Úlehlová et al. (1987). In other anatomical studies, a larger number of SG cell counts and synaptic terminals were described occasionally in the apical segment (segment 4) beyond 22 mm (Khan et al., 2005; Nadol, 1988).

In the present study, SR‐PCI revealed that the SG of the human central modiolus formed a tight spiral (450 degrees–720 degrees) supplying 9.6 mm (28%) of the total BM curvilinear length. In a 40‐year‐old individual with normal hearing, electron microscopy demonstrated 3694 cross‐sectioned myelinated peripheral axons supplying the apical turn, representing 10% of the total number of fibers innervating the cochlea (Pamulova et al., 2006). This corresponds to 4–5 mm OC length, containing around 400–500 IHCs, suggesting that each IHC in the human third turn may be innervated by 7–9 neurons. If a corresponding neural density persists in the 20–500 Hz region (9.6 mm BM length), approximately 960 IHCs would be supplied by 8152 neurons (8.5 axons/IHC). This approaches the IHC innervation density reported in the lower middle turn (Nadol, 1983; Spoendlin & Schrott, 1989). A similar number of SG cells (~8000) was obtained from our histological reconstruction (type I ganglion cells with a diameter of 22 μm filling a 1.51 mm long cylinder with a diameter of 200 μm). The data suggest that human IHCs may be more densely innervated in the apical cochlea than earlier believed. Importantly, this finding may be consistent with the apical OC's essential role in transducing low‐frequency sound to code human speech and is congruent with the results by Otte et al. (2015). A limiting factor to this finding is the equally large number of myelinated efferent fibers supplying outer hair cells in the apical cochlea and the fact that complete serial histological sectioning of the entire mid‐modiolar SG could not be accomplished in the present study.

### 4.2 Central modiolar axons are compartmentalized

An intriguing finding was the compartmentalization of central modiolar axons and dendrites within bony trabecular networks. They formed tonotopically arranged central fascicles running to the SG, the osseous spiral lamina, and OC. Regarding CI electric stimulation, this arrangement may ensure insulation of similarly tuned fibers and provide separate signal transmission despite their tightly packed arrangement beyond the first cochlear turn. This separation may be essential for low‐frequency sound resolution, tonal discrimination, and speech perception. Another notable finding was the occasional ectopic or off‐site location of functional ganglion cells. The role of these cells during CI stimulation remains speculative at present.

### 4.3 Electric stimulation of the apical cochlea

Although the SG cells were observed to remain tonotopically organized throughout the entire length, this organization and separation became increasingly compressed in the apex. In the basal turn, the angle–frequency relationship of the SG closely followed that of the OC, while above 650 degrees, the SG frequencies became much more spatially compact relative to the OC. The alignment of these angle–frequency maps in the basal turn corresponded to what was observed from the 3D segmentations, where the peripheral axons were found to follow a radial path from the BM towards the mid‐modiolar axis. In the basal turn where the OC and SG have similar angle‐frequency relationships, the frequencies decreased at a relatively steady rate of 0.1 semitones/degree. Most apically, the SG angle–frequency profile became near vertical, where several octaves of tonotopic representation spanned only 50 degrees (670 degrees–720 degrees).

Previous literature has reported improvements in speech recognition outcomes with deeper CI insertions. Specifically, speech metrics have been observed to continuously improve with angular insertion depths of up to approximately 650 degrees, which agrees with equivalent reports citing improved performance with higher coverage (Breitsprecher et al., 2023; Canfarotta et al., 2020; Dhanasingh et al., 2020; Weller et al., 2023). The improvement in performance with deeper insertions is likely attributed to increased low‐frequency tonotopic coverage. However, performance may not consistently increase with angular insertion depths greater than 700 degrees (Breitsprecher et al., 2023; Mlynski et al., 2021). This plateau may be associated with the anatomical observations of the OC and SG apical relationship presented in this work. Beyond 650 degrees, two observations were made of the SG: (1) the spatial compression of the SG tonotopy greatly increased as the SG tonotopic profile diverged from the OC; and (2) even though nerve fascicles were separated in various bone channels or trabeculae, the SG cells were contained within a shared central modiolar space without a clear bony separation between adjacent regions. Therefore, more tailored stimulation strategies may be required in the cochlear apex to maintain frequency resolution if insertion depths reach beyond 650 degrees. As the OC tonotopic map has a consistent slope through to the apical most point and does not experience the apical compression like the SG, targeted OC stimulation may allow for further improved performance with deeper angular insertion depths. In the most apical regions where SG cells are observed to be spatially compressed, rate‐based coding can provide additional frequency information to augment the tonotopic mapping.

## AUTHOR CONTRIBUTIONS

HR‐A and SA wrote the main manuscript text. All authors planned the study and analyzed the results. HL processed file data in Sweden, made 3D reconstructions, segmentation, and modeling. SA and HML prepared sample specimens and were responsible for applying SR‐PCI to image the cochlea and relevant data processing. Tonotopic mapping and analysis were made by SA, HML, and HL. HML reviewed the article, focusing on editing the methods section and was also responsible for optimizing and applying SR‐PCI to imaging of the cochlea and relevant data processing.

## CONFLICT OF INTEREST STATEMENT

MED‐EL Medical Electronics GmbH, Innsbruck, Austria provided salary support for one research group member (HLi) in accordance with the contract agreement with Uppsala University, Sweden during 2024. SA is on the surgical advisory board of MED‐EL. The funder was not, however, involved in the study design, collection, analysis, interpretation of data, the writing of this article, or the decision to submit it for publication.

## ETHICS STATEMENT

This study conforms with the Declaration of Helsinki and was approved by the Ethics Review Board (No. 99398, 22/91999, cont., 2003, no. C254/4; no. C45/72007, Dnr. 2013/190) at the Uppsala University Hospital. Written information was given to the patient, and informed consent was obtained (Tylstedt et al., 1997). The studies were conducted in accordance with the local legislation and institutional requirements. Cadaveric samples used for SR‐PCI were obtained with permission from the body bequeathal program at Western University (London, ON, Canada) in accordance with the Anatomy Act of Ontario and Western's Committee for Cadaveric Use in Research (Approval #122611). Part of the research described in this paper was performed at the Canadian Light Source, a national research facility of the University of Saskatchewan. The studies were conducted in accordance with the local legislation and institutional requirements. The participants provided written informed consent to participate in this study.

## Data Availability

Research data are not shared.

## References

[joa70001-bib-0001] Bai, S. , Encke, J. , Obando‐Leitón, M. , Weiß, R. , Schäfer, F. , Eberharter, J. et al. (2019) Electrical stimulation in the human cochlea: a computational study based on high‐resolution micro‐CT scans. Frontiers in Neuroscience, 13, 1312. 31920482 10.3389/fnins.2019.01312 PMC6915103

[joa70001-bib-0002] Bredberg, G. (1968) Cellular pattern and nerve supply of the human organ of Corti. Acta Oto‐Laryngologica, Suppl 236, 1+. 4886545

[joa70001-bib-0003] Breitsprecher, T.M. , Baumgartner, W.‐D. , Brown, K. , Dazert, S. , Doyle, U. , Dhanasingh, A. et al. (2023) Effect of Cochlear implant electrode insertion depth on speech perception outcomes: a systematic review. Otology & Neurotology Open, 3, e045. 38516541 10.1097/ONO.0000000000000045 PMC10950166

[joa70001-bib-0004] Canfarotta, M.W. , O'Connell, B.P. , Buss, E. , Pillsbury, H.C. , Brown, K.D. & Dillon, M.T. (2020) Influence of age at Cochlear implantation and frequency‐to‐place mismatch on early speech recognition in adults. Otolaryngology‐Head and Neck Surgery, 162, 926–932. 32178574 10.1177/0194599820911707 PMC8590812

[joa70001-bib-0005] Counter, S.A. , Zou, J. , Bjelke, B. , Allen Counter, S. & Klason, T. (2003) 3D MRI of the in vivo vestibulo‐cochlea labyrinth during Gd‐DTPA‐BMA uptake. Neuroreport, 14, 1707–1712. 14512842 10.1097/00001756-200309150-00010

[joa70001-bib-0006] Croner, A.M. , Heshmat, A. , Schrott‐Fischer, A. , Glueckert, R. , Hemmert, W. & Bai, S. (2022) Effects of degrees of degeneration on the electrical excitation of human spiral ganglion neurons based on a high‐resolution computer model. Frontiers in Neuroscience, 16, 914876. 35873813 10.3389/fnins.2022.914876 PMC9298973

[joa70001-bib-0007] Deman, P.R. , Van Dijk, B. , Offecier, F.E. & Govaerts, P.J. (2009) Pitch estimation of a deeply inserted cochlear implant electrode. International Journal of Audiology, 43, 363–368. 10.1080/14992020400050046 15457819

[joa70001-bib-0008] Dhanasingh, A. & Hochmair, I. (2021) Thirty years of translational research behind MED‐EL. Acta Oto‐Laryngologica, 141(Sup1), i–cxcvi. 10.1080/00016489.2021.1918399 33866929

[joa70001-bib-0009] Dhanasingh, A. , Jolly, C.N. , Rajan, G. & Van de Heyning, P. (2020) Literature review on the distribution of spiral ganglion cell bodies inside the human Cochlear central Modiolar trunk. Journal of International Advanced Otology, 16, 104–114. 32209520 10.5152/iao.2020.7510 PMC7224428

[joa70001-bib-0010] Elfarnawany, M. , Alam, S.R. , Rohani, S.A. , Zhu, N. , Agrawal, S.K. & Ladak, H.M. (2017) Micro‐CT versus synchrotron radiation phase contrast imaging of human cochlea. Journal of Microscopy, 265, 349–357. 27935035 10.1111/jmi.12507

[joa70001-bib-0011] Felder, E. , Kanonier, G. , Scholtz, A. , Rask‐Andersen, H. & Schrott‐Fischer, A. (1997) Quantitative evaluation of cochlear neurons and computer‐aided three‐dimensional reconstruction of spiral ganglion cells in humans with a peripheral loss of nerve fibres. Hearing Research, 105, 183–190. 9083815 10.1016/s0378-5955(96)00209-2

[joa70001-bib-0012] Felder, E. & Schrott‐Fischer, A. (1995) Quantitative evaluation of myelinated nerve fibres and hair cells in cochleae of humans with age‐related high‐tone hearing loss. Hearing Research, 91, 19–32. 8647720 10.1016/0378-5955(95)00158-1

[joa70001-bib-0013] Felix, H. , Johnsson, L.G. , Gleeson, M. & Pollak, A. (1990) Quantitative analysis of cochlear sensory cells and neuronal elements in man. Acta Oto‐Laryngologica. Supplementum, 470, 71–79. 2239237 10.3109/00016488909138359

[joa70001-bib-0014] Felix, H. , Johnsson, L.G. , Gleeson, M.J. , de Fraissinette, A. & Conen, V. (1992) Morphometric analysis of the cochlear nerve in man. Acta Oto‐Laryngologica, 112, 284–287. 1604993 10.1080/00016489.1992.11665419

[joa70001-bib-0015] Gani, M. , Valentini, G. , Sigrist, A. , Kós, M.‐I. & Boëx, C. (2007) Implications of deep electrode insertion on cochlear implant fitting. Journal of the Association for Research in Otolaryngology, 8, 69–83. 17216585 10.1007/s10162-006-0065-4 PMC2538415

[joa70001-bib-0016] Giese, D. , Li, H. , Liu, W. , Staxäng, K. , Hodik, M. , Ladak, H.M. et al. (2024) Microanatomy of the human tunnel of Corti structures and cochlear partition‐tonotopic variations and transcellular signaling. Journal of Anatomy, 00, 1–18. 10.1111/joa.14045 PMC11259753 38613211

[joa70001-bib-0017] Greenwood, D.D. (1961) Critical bandwidth and the frequency coordinates of the basilar membrane. The Journal of the Acoustical Society of America, 33, 1344–1356.

[joa70001-bib-0018] Guild, S.R. , Crowe, S.J. , Bunch, C.C. & Polvogt, L.M. (1931) Correlations of differences in the density of innervation of the organ of Corti with differences in the acuity of hearing, including evidence as to the location in the human cochlea of the receptors for certain tones. Acta Oto‐Laryngologica, 15, 269–308.

[joa70001-bib-0019] Hinojosa, R. , Seligsohn, R. & Lerner, S.A. (1985) Ganglion cell counts in the cochleae of patients with Normal audiograms. Acta Oto‐Laryngologica, 99, 8–13. 3976399 10.3109/00016488509119139

[joa70001-bib-0020] Hochmair, I. , Hochmair, E. , Nopp, P. , Waller, M. & Jolly, C. (2015) Deep electrode insertion and sound coding in cochlear implants. Hearing Research, 322, 14–23. 25456089 10.1016/j.heares.2014.10.006

[joa70001-bib-0021] Ishiyama, G. , Geiger, C. , Lopez, I.A. & Ishiyama, A. (2011) Spiral and vestibular ganglion estimates in archival temporal bones obtained by design based stereology and Abercrombie methods. Journal of Neuroscience Methods, 196, 76–80. 21219929 10.1016/j.jneumeth.2011.01.001

[joa70001-bib-0022] Khan, A.M. , Handzel, O. , Eddington, D.K. , Damian, D. & Nadol, J.B., Jr. (2005) Effect of cochlear implantation on residual spiral ganglion cell count as determined by comparison with the contralateral nonimplanted inner ear in humans. The Annals of Otology, Rhinology, and Laryngology, 114(5), 381–385. 15966525 10.1177/000348940511400508

[joa70001-bib-0023] Krause, W. (1876) Allgemeine und microscopische anatomie, handbuch der menschlichen anatomie, 3rd edition. Hannover: Hahn'sche Hofbuchhandlung.

[joa70001-bib-0024] Landsberger, D.M. , Mertens, G. , Punte, A.K. & van de Heyning, P. (2014) Perceptual changes in place of stimulation with long cochlear implant electrode arrays. The Journal of the Acoustical Society of America, 135, EL75–EL81. 25234918 10.1121/1.4862875 PMC3985910

[joa70001-bib-0025] Landsberger, D.M. , Vermeire, K. , Claes, A. , van Rompaey, V. & van de Heyning, P. (2016) Qualities of single electrode stimulation as a function of rate and place of stimulation with a cochlear implant. Ear and Hearing, 37, e149–e159. 26583480 10.1097/AUD.0000000000000250 PMC4844766

[joa70001-bib-0026] Lareida, A. , Beckmann, F. , Schrott‐Fischer, A. , Glueckert, R. , Freysinger, W. & Muller, B. (2009) High‐resolution X‐ray tomography of the human inner ear: synchrotron radiation‐based study of nerve fibre bundles, membranes and ganglion cells. Journal of Microscopy, 234, 95–102. 19335460 10.1111/j.1365-2818.2009.03143.x

[joa70001-bib-0027] Li, H. , Helpard, L. , Ekeroot, J. , Rohani, S.A. , Zhu, N. , Rask‐Andersen, H. et al. (2021) Three‐dimensional tonotopic mapping of the human cochlea based on synchrotron radiation phase‐contrast imaging. Scientific Reports, 11, 1–8. 33627724 10.1038/s41598-021-83225-w PMC7904830

[joa70001-bib-0028] Linthicum, F.H. & Fayad, J. (2009) Spiral ganglion cell loss is unrelated to segmental Cochlear sensory system degeneration in humans. Otology & Neurotology, 30, 418–422. 19326501 10.1097/mao.0b013e31819a8827 PMC2753358

[joa70001-bib-0029] Liu, W. , Luque, M. , Li, H. , Schrott‐Fischer, A. , Glueckert, R. , Tylstedt, S. et al. (2021) Spike generators and cell signaling in the human auditory nerve: an ultrastructural, super‐resolution, and gene hybridization study. Frontiers in Cellular Neuroscience, 15, 642211. 33796009 10.3389/fncel.2021.642211 PMC8008129

[joa70001-bib-0030] Liu, W. & Rask‐Andersen, H. (2022) GJB2 and GJB6 gene transcripts in the human cochlea: a study using RNAscope, confocal, and super‐resolution structured illumination microscopy. Frontiers in Molecular Neuroscience, 15, 973646. 36204137 10.3389/fnmol.2022.973646 PMC9530750

[joa70001-bib-0031] Miura, M. , Hirsch, B.E. , Sando, I. & Orita, Y. (2002) Analysis of spiral ganglion cell populations in children with normal and pathological ears. The Annals of Otology, Rhinology, and Laryngology, 111, 1059–1065. 12498365 10.1177/000348940211101201

[joa70001-bib-0032] Mlynski, R. , Lüsebrink, A. , Oberhoffner, T. , Langner, S. & Weiss, N.M. (2021) Mapping Cochlear duct length to electrically evoked compound action potentials in Cochlear implantation. Otology & Neurotology, 42, E254–E260. 33273309 10.1097/MAO.0000000000002957

[joa70001-bib-0033] Nadol, J. (1997) Patterns of neural degeneration in the human cochlea and auditory nerve: implications for cochlear implantation. Otolaryngology, 117, 220–228. 10.1016/s0194-5998(97)70178-5 9334769

[joa70001-bib-0034] Nadol, J.B. (1983) Serial section reconstruction of the neural poles of hair cells in the human organ of Corti. I. Inner hair cells. Laryngoscope, 93, 599–614. 6843252 10.1002/lary.1983.93.5.599

[joa70001-bib-0035] Nadol, J.B. (1988) Quantification of human spiral ganglion cells by serial section reconstruction and segmental density estimates. American Journal of Otolaryngology, 9, 47–51. 3041862 10.1016/s0196-0709(88)80007-3

[joa70001-bib-0036] Nadol, J.B. , Young, Y.S. & Glynn, R.J. (1989) Survival of spiral ganglion cells in profound sensorineural hearing loss: implications for cochlear implantation. The Annals of Otology, Rhinology, and Laryngology, 98, 411–416. 2729822 10.1177/000348948909800602

[joa70001-bib-0037] Otte, J. , Schuknecht, H.F. & Kerr, A.G. (2015) Ganglion cell populations in normal and pathological human cochleae. Implications for cochlear implantation. Laryngoscope, 125, 1038. 25801657 10.1002/lary.25219

[joa70001-bib-0038] Pamulova, L. , Linder, B. & Rask‐Andersen, H. (2006) Innervation of the apical turn of the human cochlea: a light microscopic and transmission electron microscopic investigation. Otology & Neurotology, 27, 270–275. 16437000 10.1097/01.mao.0000187239.56583.d2

[joa70001-bib-0039] Pollak, A. , Felix, H. & Schrott, A. (1987) Methodological aspects of quantitative study of spiral ganglion cells. Acta Oto‐Laryngologica, 104, 37–42. 10.3109/00016488709124974 3478959

[joa70001-bib-0040] Potrusil, T. , Heshmat, A. , Sajedi, S. , Wenger, C. , Johnson Chacko, L. , Glueckert, R. et al. (2020) Finite element analysis and three‐dimensional reconstruction of tonotopically aligned human auditory fiber pathways: a computational environment for modeling electrical stimulation by a cochlear implant based on micro‐CT. Hearing Research, 393, 108001. 32535276 10.1016/j.heares.2020.108001

[joa70001-bib-0041] Poznyakovskiy, A.A. , Zahnert, T. , Kalaidzidis, Y. , Schmidt, R. , Fischer, B. , Baumgart, J. et al. (2008) The creation of geometric three‐dimensional models of the inner ear based on micro computer tomography data. Hearing Research, 243, 95–104. 18625296 10.1016/j.heares.2008.06.008

[joa70001-bib-0042] Rask‐Andersen, H. , Li, H. , Löwenheim, H. , Müller, M. , Pfaller, K. , Schrott‐Fischer, A. et al. (2017) Supernumerary human hair cells—signs of regeneration or impaired development? A field emission scanning electron microscopy study. Upsala Journal of Medical Sciences, 122(1), 11–19. 28145795 10.1080/03009734.2016.1271843 PMC5361427

[joa70001-bib-0043] Rasmussen, A.T. (1940) Studies of the viiith cranial nerve of man. Laryngoscope, 50, 67–83.

[joa70001-bib-0044] Retzius, G. (1884) Das Gehörorgan der Wirbelthiere: morphologisch‐histologische Studien. Stockholm: Samson and Wallin.

[joa70001-bib-0045] Spoendlin, H. & Schrott, A. (1988) The spiral ganglion and the innervation of the human organ of corti. Acta Oto‐Laryngologica, 105(5–6), 403–410. 3400442 10.3109/00016488809119493

[joa70001-bib-0046] Spoendlin, H. & Schrott, A. (1989) Analysis of the human auditory nerve. Hearing Research, 43, 25–38. 2613564 10.1016/0378-5955(89)90056-7

[joa70001-bib-0047] Spoendlin, H. & Schrott, A. (1990) Quantitative evaluation of the human cochlear nerve. Acta Oto‐Laryngologica, 108, 61–70. 10.3109/00016488909138358 2239235

[joa70001-bib-0048] Tylstedt, S. , Kinnefors, A. & Rask‐Andersen, H. (1997) Neural interaction in the human spiral ganglion: a TEM study. Acta Oto‐Laryngologica, 117(4), 505–512. 9288204 10.3109/00016489709113429

[joa70001-bib-0049] Úlehlová, L. , Voldřich, L. & Janisch, R. (1987) Correlative study of sensory cell density and cochlear length in humans. Hearing Research, 28, 149–151. 3654386 10.1016/0378-5955(87)90045-1

[joa70001-bib-0050] Waldeyer, W. (1872) In: Stricker, S. (Ed.) Lehre von den Geweben des Mensch und der Thiere (Vol. II). Leipzig: verlag von Wilhelm Engelmann.

[joa70001-bib-0051] Weller, T. , Timm, M.E. , Lenarz, T. & Büchner, A. (2023) Cochlear coverage with lateral wall cochlear implant electrode arrays affects post‐operative speech recognition. PLoS One, 18(7), e0287450. 37437046 10.1371/journal.pone.0287450 PMC10337941

[joa70001-bib-0052] Wever, E. (1949) Theory of hearing. pp. 290–293.

[joa70001-bib-0053] Wright, A. (1983) Scanning electron microscopy of the human organ of Corti. Journal of the Royal Society of Medicine, 76, 269–278. 6341584 10.1177/014107688307600407 PMC1438986

[joa70001-bib-0054] Wright, A. , Davis, A. , Bredberg, G. , Ulehlová, L. , Spencer, H. , Bock, G. et al. (1987) Hair cell distributions in the normal human cochlea: a report of a European working group. Acta Oto‐Laryngologica. Supplementum, 436, 15–24. 3478958 10.3109/00016488709124972

